# Differentially expressed microRNAs targeting genes in key pathways in keratoconus

**DOI:** 10.3389/fgene.2024.1301676

**Published:** 2024-02-26

**Authors:** Dorota M. Nowak-Malczewska, Joanna Swierkowska, Marzena Gajecka

**Affiliations:** ^1^ Chair and Department of Genetics and Pharmaceutical Microbiology, Poznan University of Medical Sciences, Poznan, Poland; ^2^ Institute of Human Genetics, Polish Academy of Sciences, Poznan, Poland

**Keywords:** pre-miRNAs, microRNAs’ target genes, corneal samples, RNA-seq, extracellular matrix organization, signal transduction, miR-184

## Abstract

**Introduction:** Keratoconus (KTCN) is a corneal ectasia, characterized by a progressive thinning and protrusion of the cornea, with a complex etiology involving genetic, behavioral, lifestyle, and environmental factors. Previous studies indicated that microRNAs (miRNAs) could be involved in KTCN pathogenesis. This *in silico* study aimed to identify precursor microRNAs (pre-miRNAs) differentially expressed in KTCN corneas and to characterize mature miRNAs and their target genes.

**Materials and methods:** Expression levels of pre-miRNAs were retrieved from our previously obtained RNA sequencing data of 25 KTCN and 25 non-KTCN human corneas (PMID:28145428, PMID:30994860). Differential expression with FDR ≤0.01 and ≥1.5-fold changes were considered significant. Lists of target genes (target score ≥90) of mature miRNAs were obtained from miRDB. Revealed up-/downregulated miRNAs and their target genes were assessed in databases and literature. Enrichment analyses were completed applying the DAVID database.

**Results:** From a total of 47 pre-miRNAs, six were remarkably upregulated (*MIR184*, *MIR548I1*, *MIR200A*, *MIR6728*, *MIR429*, *MIR1299*) and four downregulated (*MIR6081*, *MIR27B*, *MIR23B*, *MIR23A*) in KTCN corneas. Out of the 1,409 target genes, 220 genes with decreased and 57 genes with increased expression levels in KTCN samples vs non-KTCN samples were found. The extracellular matrix (ECM) organization, response to mechanical stimulus, regulation of cell shape, and signal transduction processes/pathways were identified as distinctive in enrichment analyses. Also, processes associated with the regulation of transcription and DNA binding were listed.

**Conclusion:** Indicated miRNAs and their target genes might be involved in KTCN pathogenesis via disruption of crucial molecular processes, including ECM organization and signal transduction.

## 1 Introduction

Keratoconus (KTCN) is a corneal ectasia characterized by a progressive thinning and anterior protrusion of the cornea leading to reduced vision and changed refractive power (Reinstein et al., 2015). KTCN is typically bilateral but asymmetrical, with an onset usually occurring in late adolescence or early adulthood. KTCN is a multifactorial disease with a complex etiology involving multiple genes, as well as behavioral, lifestyle, and environmental factors (Karolak and Gajecka, 2017; Jaskiewicz et al., 2023c). Previously, we have performed RNA sequencing (RNA-seq) on corneas derived from 25 Polish patients with KTCN and 25 control individuals and revealed disruption of collagen synthesis and downregulation of core elements of the TGF-β, Hippo, and Wnt pathways in KTCN corneas (Kabza et al., 2017b). Then, we performed reanalysis of the RNA-seq dataset and have indicated 12 downregulated and 6 upregulated genes in KTCN that overlapped or were located in the near vicinity of the identified differentially methylated regions (Kabza et al., 2019). Moreover, analysis of the KTCN transcriptome allowed us to identify long non-coding RNAs that might be involved in KTCN etiology (Szczesniak et al., 2017).

Here, to complement both our *in silico* analysis of microRNAs (miRNAs) (Nowak and Gajecka, 2015) and our experimental transcriptomic study on KTCN and non-KTCN corneas (Kabza et al., 2017b; 2019), we retrieved differentially expressed precursor miRNAs (pre-miRNA) from our RNA-seq data. Mentioned miRNAs belong to a highly conserved class of small non-coding RNAs, about 22 nucleotides in length, which play substantial roles in regulating gene expression. The majority of miRNAs are transcribed from miRNA encoding genes by a RNA polymerase II to form primary miRNAs (pri-miRNAs). Then nuclear RNase III enzyme Drosha and its cofactor process pri-miRNAs into 60 nt length precursor miRNAs (pre-miRNAs) and further processing by RNase III enzyme Dicer leads to creation of mature miRNAs (Ghorai and Ghosh, 2014; Fuchs Wightman et al., 2018). Previously, miRNAs have been studied in KTCN mainly with the focus on miR-184 that was reported to be related to this disease (Hughes et al., 2011; Lechner et al., 2013; Bykhovskaya et al., 2015; Farzadfard et al., 2016; Stachon et al., 2022; Zhang et al., 2022).

The profile of miRNAs in KTCN has been already studied, however, there are substantial sequence variation and gene expression differences between populations and even families with KTCN (Karolak et al., 2011; 2016; Czugala et al., 2012), so the identified features characteristic for Polish patients, may, but do not have to, be a repetition of the already published results. As far as we are concerned, the miRNA profile in the corneas of KTCN patients from the Polish population has never been published. Thus, the purpose of our *in silico* study was to identify differentially expressed pre-miRNAs, and characterize mature miRNAs, their target genes and enriched molecular processes and signaling pathways to indicate those involved in KTCN pathogenesis.

## 2 Materials and methods

### 2.1 Corneal samples and RNA sequencing data

Transcriptomic data from previously performed RNA-seq of 25 KTCN and 25 non-KTCN human corneas (Kabza et al., 2017b; Kabza et al., 2017a) was assessed. The 25 KTCN corneal tissues were derived from non-related Polish patients undergoing a keratoplasty procedure. The control 25 non-KTCN corneas were collected from patients, who were referred for corneal transplantation due to other reasons, such as bullous keratopathy, corneal scarring, perforations and ulcers. Medical examination of patients, clinical metadata, the handling of the corneas, and RNA extraction have been described elsewhere (Kabza et al., 2017b).

RNA-seq data was originally analyzed in 2017 (Kabza et al., 2017b) and then underwent reanalysis with the use of a modified pipeline in 2019 (Kabza et al., 2019). In the current study the data of the reanalysis (Kabza et al., 2019) was used.

### 2.2 Assessment of differential expression

Expression data for pre-miRNA was retrieved from the RNA-seq datasets that have been deposited in the Gene Expression Omnibus with the accession number GSE77938. An edgeR-limma workflow was employed to analyze RNA-seq data, utilizing gene-level counts as input (Robinson et al., 2010; Ritchie et al., 2015). The workflow encompasses pre-processing and exploratory data analysis, culminating in identifying differentially expressed genes and gene signatures. The analysis adhered to a previously established protocol (Law et al., 2016). Genes were deemed differentially expressed if they met the criteria of a false discovery rate (FDR) threshold value of 0.01 and a fold change of ≥1.5. The detailed bioinformatics workflow for this analysis was described in our previous publication (Kabza et al., 2019).

### 2.3 Assessment of miRNA encoding genes

Differentially expressed miRNA encoding genes were searched in miRBase^1^, Mouse Genome Informatics (MGI)^2^, and available literature data.

To assess sequence variation in miRNA encoding genes, variants derived from Ensembl exons were called by FreeBayes (Garrison and Marth, 2012). The obtained DNA sequence variants were filtered according to the Utah Genome Project Variant Calling Protocol (Garrison and Marth, 2012) and compared to a list of pre-miRNA coordinates by an in-home Python script.

### 2.4 Prediction of miRNA’s targets

An online database miRDB^3^ (Liu and Wang, 2019), containing predicted miRNAs’ targets in a human, was searched. Target genes were identified using the MirTarget2 algorithm, developed through the analysis of thousands of genes regulated by miRNAs using the support vector machines (Liu and Wang, 2019). A target score between 50 and 100 is assigned to each of the probable target genes, with higher scores indicating more reliable predictions of binding sites within the given genes for each miRNA (Liu and Wang, 2019). A score of ≥80, indicating the most likely real target genes, is recommended in the miRDB. Here, binding sites with a score value of 90 or greater were selected for further analysis to increase the prediction reliability.

### 2.5 Pathway enrichment analysis

The Kyoto Encyclopedia of Genes and Genomes (KEGG), REACTOME and Gene Ontology (GO) analyses were conducted applying the Database for Annotation, Visualization, and Integrated Discovery (DAVID) (Huang et al., 2009; Sherman et al., 2022). Genes with statistically significant increase and decrease in expression level were subjected to separate analyses in the aspect of serving as the targets for the analyzed miRNAs. This approach allowed for an exploration of the regulatory interactions between miRNAs and their corresponding target genes, shedding light on the mechanisms underlying gene expression in the cornea. The focus was given on biological process (BP), molecular function (MF), cellular component (CC) and KEGG/REACTOME pathways. *p*-value <0.05 and number of genes for each of identified GO terms/processes/pathways ≥3 were considered as the cutoff criteria in GO, REACTOME, and KEGG analyzes.

The obtained results were also compared to the results from our previously performed overrepresentation analysis of molecular pathways among genes upregulated and downregulated in KTCN (Kabza et al., 2017b).

## 3 Results

### 3.1 Differential expression of miRNA precursors

A total of 47 pre-miRNAs in the studied corneal samples were identified (Supplementary Table S1). Six pre-miRNAs were found to be significantly upregulated, while four were significantly downregulated (Table 1), comparing KTCN vs non-KTCN samples. Those 10 pre-miRNAs are processed by Dicer into a total of 15 mature miRNAs, as five of these precursors form miRNAs from either the 3′or 5′end, as indicated in Table 1. Upregulation or downregulation of a pre-miRNA was indicated by the value of logFC. The pre-miRNA with the most altered expression was hsa-miR-184, which was found to be upregulated.

**TABLE 1:** Differentially expressed pre-miRNAs in KTCN corneas vs non-KTCN corneas**.** Upregulation and downregulation of miRNA is indicated by the value of logFC.

Gene	logFC	Fold change	*p*-value	FDR	Mean expression in KTCN corneas	Mean expression in non-KTCN corneas	t	B	Gene	*Locus*	Precursor miRNA	Mature miRNA
Upregulated miRNAs precursors												
ENSG00000207695	1.52	2.88	3.78E-06	4.45 × 10^−5^	559.43	241.61	5.18	4.20	*MIR184*	15:79209788–79209871(+)	hsa-mir-184	hsa-miR-184
ENSG00000221737	1.22	2.33	2.06E-04	1.13 × 10^−3^	20.23	7.76	3.99	0.60	*MIR548I1*	3:125790404–125790552(−)	hsa-mir-548i-1	hsa-miR-548i
ENSG00000207607	1.22	2.33	4.76E-05	3.42 × 10^−4^	44.86	17.06	4.44	1.82	*MIR200A*	1:1167863–1167952(+)	hsa-mir-200a	hsa-miR-200a-5p, hsa-miR-200a-3p
ENSG00000274258	1.15	2.22	2.63E-05	2.10 × 10^−4^	48.12	23.15	4.62	2.40	*MIR6728*	1:8866502–8866590(−)	hsa-mir-6728	hsa-miR-6728-5p, hsa-miR-6728-3p
ENSG00000198976	0.74	1.67	6.90E-04	3.09 × 10^−3^	66.35	34.65	3.61	−0.48	*MIR429*	1:1169005–1169087(+)	hsa-mir-429	hsa-miR-429
ENSG00000275377	0.72	1.64	1.13E-03	4.65 × 10^−3^	219.26	123.94	3.45	−1.10	*MIR1299*	9:40929010–40929092(−)	hsa-mir-1299	hsa-miR-1299
Downregulated miRNAs precursors												
ENSG00000274115	−0.94	1.92	4.82E-05	3.45 × 10^−4^	34.94	62.97	−4.44	1.91	*MIR6081*	9:95065350–95065446(+)	hsa-mir-6081	miR-6081
ENSG00000207864	−0.70	1.62	8.44E-05	5.41 × 10^−4^	72.70	114.82	−4.27	1.33	*MIR27B*	9:95085445–95085541(+)	hsa-mir-27b	hsa-miR-27b-5p, hsa-miR-27b-3p
ENSG00000207563	−0.63	1.54	1.44E-03	5.67 × 10^−3^	25.68	41.18	−3.37	−1.14	*MIR23B*	9:95085208–95085304(+)	hsa-mir-23b	hsa-miR-23b-5p, hsa-miR-23b-3p
ENSG00000207980	−0.60	1.52	2.36E-03	8.56 × 10^−3^	60.31	84.10	−3.20	−1.62	*MIR23A*	19:13836587–13836659(−)	hsa-mir-23a	hsa-miR-23a-5p, hsa-miR-23a-3p

LogFC, logarithmic fold change; FDR, false discovery rate; t - t-statistic; B- B-statistic.

### 3.2 Characterization of miRNA encoding genes

Of the analyzed pre-miRNAs, four are located intra-intronic. *MIR6728* is located in the intron of the enolase 1 (*ENO1*) gene at chromosome 1. The mean expression of *ENO1* was 2,649.65 transcripts per million (TPM) in KTCN corneas, and 1,251.51 TPM in non-KTCN corneas (2.06-fold change, FDR *p*-value = 2.47 × 10^−7^). *MIR6081*, *MIR27B*, and *MIR23B* genes (with levels of expression presented in Table 1) are located in introns of the aminopeptidase O gene (putative, *AOPEP,* expression level: 53.10 TPM in KTCN vs. 75.47 TPM in non-KTCN corneas) at chromosome 9. In addition, four genes *MIR184*, *MIR548I1*, *MIR200A* and *MIR429* (with levels of expression shown in Table 1) are located within genes encoding lncRNAs, including *ANKRD34C-AS1*, *LOC105374312*, *LOC124903818* and again in *LOC124903818*, respectively.

Searching the MGI database, we found that *MIR23B*, and *MIR23A*, were expressed in murine eyes at low and/or medium levels. *MIR200A* was found to be expressed at low level in RNA-seq, *MIR184* and *MIR27B* were detected in RNA *in situ*/RT-PCR but not in RNA-seq, and *MIR429* has not been expressed. All the mentioned MGI data is available at the following accession numbers: E-GEOD-33141, E-GEOD-63810, E-MTAB-6133, E-MTAB-9143; J:190177, J:124661, J:151925, J:124661, J:310450. There was no data available in the MGI for *MIR548I1*, *MIR6728*, *MIR1299*, and *MIR6081* genes.

Assessing the DNA sequence variation, no variants were found within genes encoding the evaluated pre-miRNAs.

### 3.3 Target genes of differentially expressed miRNAs

We further investigated the potential target genes (listed in Supplementary Table S2) for the selected 15 mature miRNAs (listed in Table 1, in the last column) using the miRDB database, and a total of 1,911 interactions were identified (Supplementary Table S2). Of these, 420 genes were regulated by more than one of the identified miRNAs, with five genes being targets of four of the miRNAs (*TMEM135*, *DTNA*, *NDFIP2*, *NCOA2*), 76 genes being targets of three miRNAs, and 339 genes being targets of two miRNAs. However, not all of these genes exhibited statistically significant altered expression levels. Among the genes regulated by these four miRNAs (miR-429, miR-27b-3p, miR-23b-3p, miR-23a-3p), only *DTNA* showed a statistically significant decrease in expression within the studied KTCN corneas. Specifically, the precursor coding miR-429 was detected with increased expression level, while the precursors for miR-27b-3p, miR-23b-3p, and miR-23a-3p displayed decreased expression levels in KTCN corneas.

**FIGURE 1:**
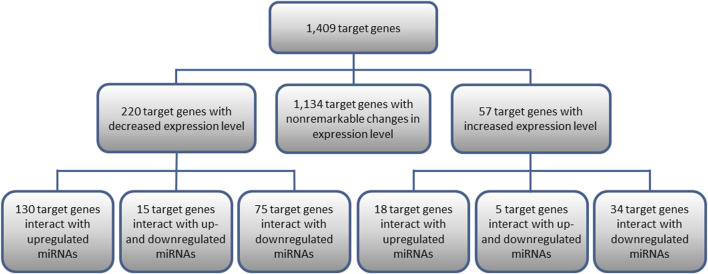
Target genes of 10 differentially expressed pre-miRNAs and their interactions with miRNAs. Presented are target genes with decreased or increased expression levels found to be statistically significant in KTCN corneas compared to non-KTCN corneas.

Out of the 1,409 genes regulated by the analyzed miRNAs, 220 genes with significantly decreased expression and 57 with increased expression levels in KTCN samples were identified comparing KTCN to non-KTCN samples. Out of the 220 target genes displaying significantly decreased expression levels, 130 genes were found to interact with upregulated miRNAs, 75 genes with downregulated miRNAs, and 15 genes with both up- and downregulated miRNAs (Figure 1). Also, out of the 57 genes displaying increased expression, 18 genes were found to interact with upregulated miRNAs, 34 genes with downregulated miRNAs, and five genes with both up- and downregulated miRNAs (Figure 1).

### 3.4 Enriched molecular processes and signaling pathways

The enrichment analysis of miRNA’s target genes was conducted using the DAVID database and encompassed 220 genes with decreased and 57 with increased expression levels in KTCN corneas. Specifically, the downregulated genes were associated with 97 GO BP terms, 34 GO CC terms, and 30 MF terms. Furthermore, they were categorized into 24 processes/pathways within the KEGG database and 30 processes/pathways within the REACTOME database. Similarly, the upregulated target genes were linked to four GO BP terms, four GO CC terms, one KEGG pathway, and one REACTOME pathway (Supplementary Table S3).

**FIGURE 2:**
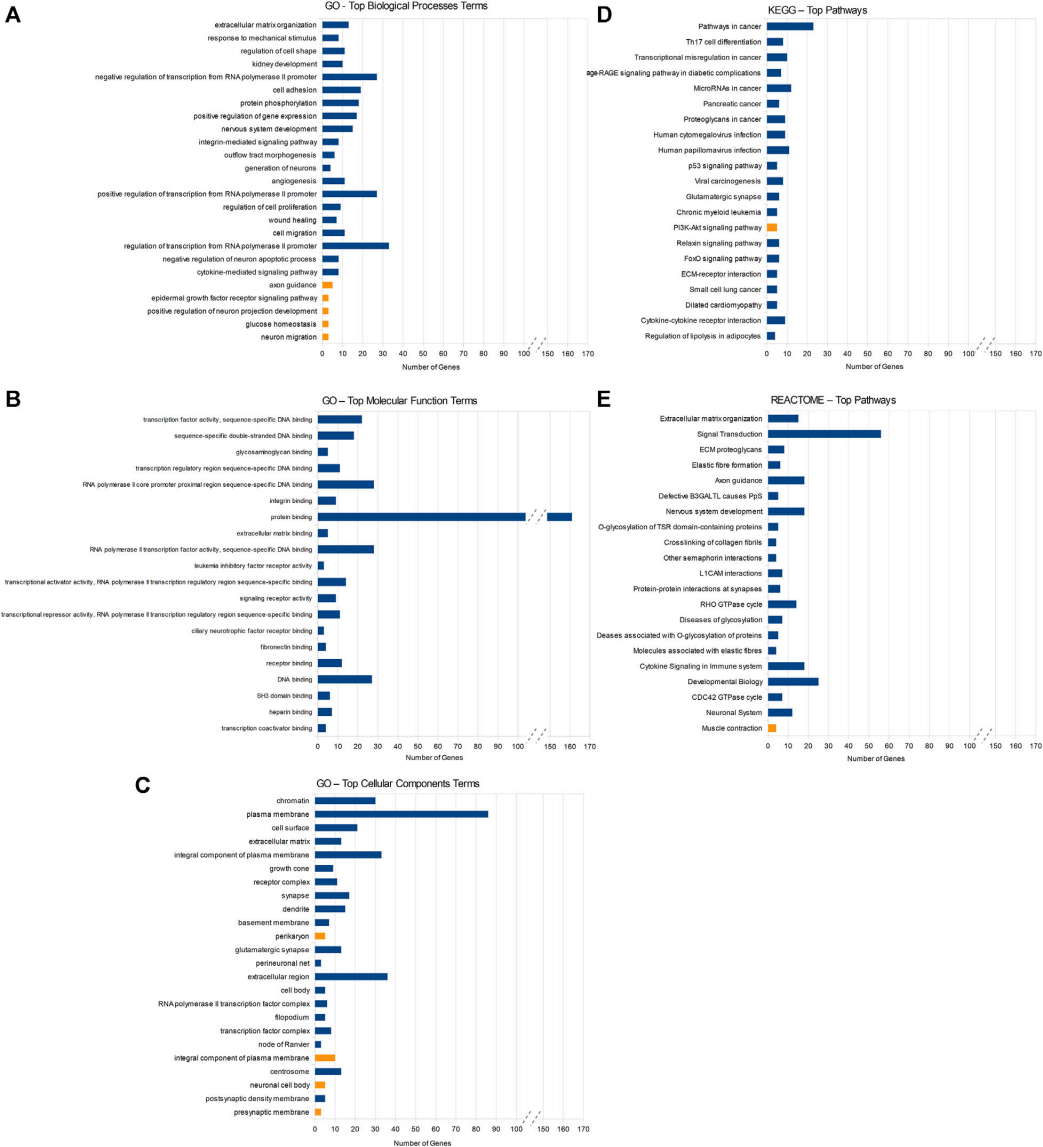
Results of DAVID enrichment analyses of miRNAs’ target genes in different databases: Gene Ontology (GO) for biological processes **(A)**, molecular function **(B)**, cellular component **(C)**, the Kyoto Encyclopedia of Genes and Genomes (KEGG) **(D)**, and REACTOME **(E)**. Blue rectangles indicate pathways enriched with target genes of downregulated miRNAs and yellow rectangles stand for pathways enriched with target genes of upregulated miRNAs. Statistical significance decreases from top to bottom in all the figures.

Among the downregulated target genes, the three GO BP terms showing the lowest *p*-values were the ECM organization (13 genes, *p*-value = 5.95 × 10^−7^), response to mechanical stimulus (8 genes, *p*-value = 2.03 × 10^−6^), and regulation of cell shape (11 genes, *p*-value = 3.96 × 10^−6^). The GO MF indicated mainly molecular processes associated with the regulation of transcription and DNA binding (Figure 2). The REACTOME analysis pointed towards pathways associated with the ECM organization (15 genes, *p*-value = 3.38 × 10^−5^) and signal transduction (56 genes, *p*-value = 3.85 × 10^−5^). Compared to downregulated genes, fewer processes/pathways involving upregulated target genes were identified (Figure 2).

We also compared the current data and results to the outcomes from our previous pathway overrepresentation analysis of the whole corneal transcriptome. In both, the analysis of the entire KTCN transcriptome and the analysis of the target genes for the miRNAs only, the majority of genes was assigned to the signal transduction pathway (Supplementary Table S4). Out of the genes found in the prominent pathways identified in the whole-transcriptome analysis, 34 genes exhibited regulation by the miRNAs assessed here. Among the significant elements in previously discussed pathways (TGF-β, Hippo, and Wnt pathways), two genes (*TGFBR2* and *FZD7*) with decreased expression in KTCN corneas are controlled by miRNAs, whose precursors showed altered expression. *TGFBR2* is a potential target gene of miR-23b-3p and miR-23a-3p, for which we observed decreased precursor expression in the analyzed samples. Whereas *FZD7* is a target gene of miR-548i, which precursor exhibited significantly increased expression level.

## 4 Discussion

We identified six significantly upregulated and four downregulated in KTCN pre-miRNAs in the RNA-seq data of the studied corneal samples derived from patients with KTCN and controls. The highest difference in expression between KTCN and non-KTCN corneas was reached by the upregulated miR-184, previously reported to be involved in KTCN and other corneal abnormalities (Hughes et al., 2011; Lechner et al., 2013; Bykhovskaya et al., 2015; Farzadfard et al., 2016). This miRNA has been found to be one of the most abundant miRNAs in the cornea and expressed in the corneal epithelium and stroma (Abu-Amero et al., 2015). The miR-184 regulates the expression of genes involved in cell proliferation, differentiation, and apoptosis, suggesting that it is a key regulator of corneal development and homeostasis (Abu-Amero et al., 2015). Previously, the c.57 C > T mutation in miR-184 has been identified to be responsible for inherited corneal and lens abnormalities in a five-generation family from Spain and in an autosomal-dominant form of severe anterior KTCN and early-onset anterior polar cataract in a Northern Irish family (Hughes et al., 2011; Bykhovskaya et al., 2015). On the contrary, in our miRDB search we identified only three miR-184 target genes with scores ≥90, namely, *SETD9*, *NUS1*, and *CES2*, and despite the considerable upregulation of pre-miR184, none of these genes showed altered expression in the analyzed samples. Therefore, upregulation of miR-184 might not have played a crucial role in the KTCN pathogenesis in our patients, especially that we found no sequence variants in the seed region of the *MIR184* gene. Also no mutations were reported in the miR-184 seed region in Saudi Arabian patients with KTCN (Abu-Amero et al., 2015) and no association with sporadic KTCN in Greek patients has been revealed in genotyping of rs41280052 located in *MIR184* (Moschos et al., 2016). It is worth emphasizing that although in our *in silico* analyses no target genes with altered expression for miR-184 were indicated, there are various genes that may be regulated by this miRNA. An example of such a gene is *FZD7*, which regulation by miRNA and, consequently, its impact on retinal neovascularization through the Wnt pathway, has been studied by Takahashi et al. (Takahashi et al., 2015). In our transcriptomic data, a significant decrease in *FZD7* expression was observed in KTCN patients. It is possible that the increased level of mir-184 affects the Wnt pathway by regulating *FZD7* expression. Therefore, it is important to consider the limitations of computer-based analyses, which may not always provide a comprehensive understanding of the investigated issue.

The identified *MIR6728*, *MIR23B*, *MIR27B*, and *MIR6081* miRNA genes encode intronic miRNA that are transcribed together with *ENO1* (*MIR6728*) and *AOPEP* (*MIR23B*, *MIR27B*, *MIR6081*) genes. *MIR6728* is located in the *locus* 1p36.23–36.21 that has been identified in the linkage analysis in an Australian KTCN family (Burdon et al., 2008) and has been also discussed in our previous paper (Nowak and Gajecka, 2015). The *ENO1* gene encodes enolase 1 (α-enolase) that functions as a glycolytic enzyme and also as a structural lens protein (tau-crystallin) in the monomeric form. In a previous study by Srivastava et al., relatively low or negligible levels of α-enolase and β-actin were detected in the epithelial cells of KTCN corneas compared to the normal levels observed in non-KTCN corneas (Srivastava et al., 2006). On the contrary, in our study the transcript of *ENO1* reached a fold change of 2.06 with the higher expression level in KTCN corneas vs non-KTCN corneas. On the other hand, in the study by Burdon et al. no mutations were identified in *ENO1* (Burdon et al., 2008). Statistically significant increased expression was also observed for the *MIR6728* gene located within *ENO1*. Furthermore, we detected remarkable changes in the expression of target genes controlled by miR-6728-5p and/or miR-6728-3p, including the *LOX* gene, which is widely discussed in the context of KTCN. *LOX* encodes lysyl oxidase, an enzyme responsible for catalyzing the cross-linking of collagen and elastin fibers in the ECM. Previous studies have indicated that *LOX* expression is often decreased in the corneal tissue of individuals with KTCN (Dudakova et al., 2012; Shetty et al., 2015; Takaoka et al., 2016). Reduced LOX activity can lead to insufficient cross-linking of collagen and elastin fibers in the cornea, which in turn can result in corneal weakening and thinning (Dudakova et al., 2012). Our analyzes suggest that increased *MIR6728* level, which encodes miR-6728-5p, might result in a substantial downregulation of *LOX* expression.

The second gene with an intronic miRNA encoding gene is *AOPEP* that encodes aminopeptidase O (putative) and is a member of the M1 zinc aminopeptidase family. *MIR27B* and *MIR23B* are in the miR-23b/27b/24–1 cluster. Two miR-23/27/24 clusters exist in the vertebrate genome: an intergenic miR-23a/27a/24–2 cluster and an intronic miR-23b/27b/24–1 cluster (Zhou et al., 2011). MiRNAs encoded by the miR-23/27/24 gene clusters are enriched in endothelial cells and highly vascularized tissues. Interestingly, one of our indicated miRNA encoding genes, *MIR6081*, is localized in a close proximity to this cluster. Inhibition of miR-23 and miR-27 represses angiogenesis *in vitro*, postnatal retinal vascular development and choroidal neovascularization in response to laser injury in a mouse model (Zhou et al., 2011). MiR-23 and miR-27 enhance angiogenesis by promoting angiogenic signaling through targeting Sprouty2 and Sema6A proteins (Zhou et al., 2011). Previously, expression levels of miR-23a-3p and miR-23b-3p have been found to be significantly upregulated in patients with senile cataract when compared with the control group (Kim et al., 2021). Overexpression of miR-23b-3p also promoted the proliferation, migration, and epithelial-mesenchymal transition of lens epithelial cells by targeting Sprouty2 (Liu et al., 2019). Furthermore, under oxidative stress, when the activation of apoptosis of lens epithelial cells may lead to the opacity of the lens and accelerate the progression of cataract, miR-23b-3p regulated apoptosis and autophagy via suppressing SIRT1 in these cells (Zhou et al., 2019). It has been indicated that miR-23a protects retinal pigment epithelium cells against oxidative damage through regulation of Fas cell surface death receptor or glutaminase-1 and glutamine uptake (Lin et al., 2011; Li et al., 2016; Zhou et al., 2020). High gene copy number of miR-23a was also associated with susceptibility to acute anterior uveitis (Yang et al., 2017). Then, miR-27b inhibited adipogenesis in orbital fibroblasts from patients with Graves’ orbitopathy (Jang et al., 2019). However, we did not observe changes in expression level of the mentioned *SEMA6A*, *SPRY2*, and *SIRT1* genes in our data.

Considering other identified miRNAs, miR-429 plays an important role in hypoxia-induced retinopathy by inhibiting the HPSE-VEGF pathway and was downregulated in pterygium (Engelsvold et al., 2013; Xu H. et al., 2022). Also, members of the miR-200 family, including miR-200a, were downregulated and could be potential regulators of epithelial-mesenchymal transition in pterygium (Engelsvold et al., 2013). Also, miR-200a could play a protective role in the glaucoma-induced optical nerve injury by inhibiting the FGF7-mediated MAPK signaling pathway (Peng et al., 2019).

Performed pathway enrichment analyses highlighted the significance of our previously obtained results concerning the processes and signaling pathways involved in KTCN pathogenesis. Enrichment analyses of downregulated target genes pointed to several molecular processes and signaling pathways with the highest implication for ECM organization, response to mechanical stimulus, regulation of cell shape, and signal transduction. Also, GO terms enrichment analysis indicated mainly molecular processes associated with the regulation of transcription processes and DNA binding were revealed in the GO assessment. This is in line with our previous studies on KTCN corneas as we had identified differentially expressed genes that were involved in ECM organization, and signal transduction, including Wnt signaling pathways, disrupted in KTCN, assessing transcriptomic (Kabza et al., 2017b; Karolak et al., 2020b), genomic (Karolak et al., 2020a), and mitochondrial features (Nowak-Malczewska et al., 2021). Moreover, in our continuous study on additional corneal samples derived again from unrelated Polish patients with KTCN, we have identified both coding and non-coding sequence variation in genes that are involved, among others, in ECM and Wnt signaling (Jaskiewicz et al., 2023a). Cell-ECM interactions and ECM processing, being the critical elements of the wound healing process, were altered in corneal topographic regions of corneal epithelium from 23 patients with KTCN (Jaskiewicz et al., 2023b). Furthermore, we identified changes in DNA methylation level in *WNT3* and *WNT5A* genes encoding Wnt ligands, which might be an explanation for the Wnt signaling pathway dysregulation in KTCN (Kabza et al., 2019). Several other studies support our results (Khaled et al., 2018; Xu L. et al., 2022; Dou et al., 2022; Akoto et al., 2023), including Bykhovskaya et al. claiming that the development of KTCN results in deregulation of gene expression of ECM and adhesion molecules (Bykhovskaya et al., 2016). The result highlighting the pathway of response to mechanical stimuli is consistent with the existing literature and our current studies on environmental factors and habits of patients with KTCN, which have pointed to repetitive mechanical corneal injuries caused by eye rubbing as a factor contributing to the development of KTCN, potentially through the alteration of gene expression in corneal epithelium (Najmi et al., 2019; Jaskiewicz et al., 2023c).

The limitation of our study is that the project was based on the RNA-seq data instead of the data of the small RNA sequencing. Thus, we analyzed expression levels of pre-miRNAs instead of the experimentally obtained mature miRNAs. Further experimental studies, including functional analyses were beyond the scope of this project. It remains possible that KTCN development might have led to changes in the expression level of these miRNAs and not the opposite. One possibility is the influence of the impaired wound healing process on the observed changes in pre-miRNA expression and, consequently, their influence on processes that are crucial in the development of KTCN.

Summarizing, in this study on miRNAs in corneas of KTCN patients from the Polish population we identified pre-miRNAs expressed in KTCN corneas and characterized *in silico* the mature miRNAs, their target genes and molecular processes/signaling pathways that could be involved in KTCN pathogenesis. The identified differentially expressed miRNAs might contribute to KTCN pathogenesis via disruption of ECM organization and signal transduction pathways that we have already indicated as causative in our previous KTCN research.

## Data Availability

The original contributions presented in the study are included in the article/Supplementary Material, further inquiries can be directed to the corresponding author.

## Footnotes

1
https://www.mirbase.org/

2
http://www.informatics.jax.org/

3
http://mirdb.org/

## Abbreviations

BP, biological process; CC, cellular component; ECM, extracellular matrix; FDR, false discovery rate; KTCN, keratoconus; MF, molecular function; miRNAs, microRNAs; pre-miRNAs, precursor miRNAs; pri-miRNAs, primary miRNAs; TPM, transcripts per million.
